# Appreciation to Reviewers in 2020

**DOI:** 10.1055/s-0041-1732445

**Published:** 2021-10-04

*AORTA* would like to thank the following reviewers for their contribution and support as expert peer reviewers of submitted scientific papers in 2020. Your efforts, insight, and judgment are greatly appreciated. Thank you for your contributions to *AORTA* in 2020.

Donald Adam

Daniel Anderson

Roland Assi, MD, MMS

Simon C. Body, MBChB, MPH, FAHA

Michael A. Borger, MD, PhD

Oliver Bouchot, MD, PhD

Harisios Boudoulas, MD

Alan Braverman, MD

Duke Cameron

Ludovic Canaud, MD, PhD

Davide Carino, MD

Thierry Carrel, MD

Roberto Chiesa

Lawrence S. Cohen, MD

Sitaram Chilakamarry

Anneke Damberg, MD

Alan Dardik, MD, PhD

Luca Di Marco, MD

Alan Daugherty, PhD, DSc

Tirone E. David

Abe DeAnda, MD

Dimitrios Dougenis, MD, PhD

Roberto Di Bartolomeo

Julia Dumfarth, MD

Young Erben, MD

Emily Farkas

Theodor Fischlein

Anthony Furnary, MD

S. David Gertz, MD, PhD

Leonard Girardi, MD

Robin Heijmen, MD, PhD

Sarah Hull

Jay Humphrey

Gabriele Iannelli, MD

John Ikonomidis, MD, PhD

Jeffrey Indes, MD

Kim de la Cruz, MD

Ion Jovin, MD

Paris Kalogerakos, MD, PhD

Habib Khan, MBBS

Ulas Kumbasar, MD

Scott A. LeMaire, MD

George V. Letsou, MD

Robert Levine, MD

Yupeng Li, PhD

Wei-Guo Ma, MD, PhD

Giovanni Mariscalco

Jorge G. Mascaro, MD

Bernardo Mendes

D. Craig Miller

Salvior MOK, MD

Makoto Mori, MD

Giacomo Murana

Spyros Mylonas, MD

Aung Oo, MD

Kristine Clodfelter Orion, MD

Philip Pang, MD

Sven Peterss, MD

Gabriele Piffaretti, MD, PhD

Ourania Preventza, MD

Cha Rajakaruna, MD

T. Brett Reece

John Rizzo

Charles Roberts

Chris Rokkas

Stefano Schena, MD, PhD

Ulrich Schneider

Prem Shekar, MD, MBA

Konstantinos Spanos

Louis Stein, MD, PhD, MHS

Wei Sun

Hiroo Takayama, MD, PhD

Ramesh K. Tripathi, MD, FRCS, FRACS

Marko Turina, MD

Gilbert R. Upchurch, MD

Konstantin von Aspern, MD

Michiel Vriesendorp, MD

Bulat A. Ziganshin, MD, PhD

Bijoy G. Rajbanshi, MD

